# Menstrual Cycle and Progesterone‐to‐Estradiol Ratio Associated With Loss of Control and Craving in Alcohol Use Disorder: A Sex‐Segregating Longitudinal Study

**DOI:** 10.1111/adb.70192

**Published:** 2026-10-04

**Authors:** Sabine Hoffmann, Iris Reinhard, Christiane Mühle, Patrick Bach, Leonard Wenger, Dominic Reichert, Rafat Boroumand‐Jazi, Sibel Nayman, Christine Kuehner, Matthias Reichl, Anne Koopmann, Anne Beck, Hilmar G. Zech, Johannes Kornhuber, Christian P. Müller, Rajita Sinha, Wolfgang H. Sommer, Rainer Spanagel, Tobias Banaschewski, Lorenz Deserno, Ulrich W. Ebner‐Priemer, Andreas Meyer‐Lindenberg, Michael A. Rapp, Stephanie H. Witt, Michael N. Smolka, Andreas Heinz, Heike Tost, Falk Kiefer, Markus Reichert, Bernd Lenz, for the ReCoDe‐Consortium

**Affiliations:** ^1^ Department of Addictive Behavior and Addiction Medicine, Central Institute of Mental Health (CIMH), Medical Faculty Mannheim Heidelberg University Mannheim Germany; ^2^ German Center for Mental Health, Partner Site Mannheim‐Heidelberg‐Ulm Mannheim Germany; ^3^ Core Facility Biostatistics, Central Institute of Mental Health (CIMH), Medical Faculty Mannheim Heidelberg University Mannheim Germany; ^4^ Department of Psychiatry and Psychotherapy Friedrich‐Alexander University Erlangen‐Nürnberg (FAU) Erlangen Germany; ^5^ Department of Psychiatry and Psychotherapy, Central Institute of Mental Health (CIMH), Medical Faculty Mannheim Heidelberg University Mannheim Germany; ^6^ Department for Sport and Exercise Science Paris Lodron University Salzburg Salzburg Austria; ^7^ Department of Psychosomatic Medicine and Psychotherapy, Central Institute of Mental Health, Medical Faculty Mannheim Heidelberg University Mannheim Germany; ^8^ Department of Neuropsychology and Psychological Resilience Research, Central Institute of Mental Health, Medical Faculty Mannheim Heidelberg University Mannheim Germany; ^9^ Institute for Mental Health and Behavioral Medicine, Department of Psychology HMU Health and Medical University Potsdam Potsdam Germany; ^10^ Department of Psychiatry and Psychotherapy Technische Universität Dresden Dresden Germany; ^11^ Department of Child and Adolescent Psychiatry, Psychotherapy and Psychosomatics University of Würzburg Würzburg Germany; ^12^ Institute of Psychopharmacology, Central Institute of Mental Health (CIMH), Medical Faculty Mannheim Heidelberg University Mannheim Germany; ^13^ Yale Stress Center, Department of Psychiatry Yale University School of Medicine New Haven Connecticut USA; ^14^ Bethanian Hospital for Psychiatry, Psychosomatics and Psychotherapy Greifswald Germany; ^15^ Department of Child and Adolescent Psychiatry and Psychotherapy, Central Institute of Mental Health (CIMH), Medical Faculty Mannheim Heidelberg University Mannheim Germany; ^16^ Mental mHealth Lab, Department of Sports and Sports Science Karlsruhe Institute of Technology (KIT) Karlsruhe Germany; ^17^ Department of Social and Preventive Medicine University of Potsdam Potsdam Germany; ^18^ German Center for Mental Health, Partner Site Berlin‐Potsdam Berlin Germany; ^19^ Department of Genetic Epidemiology in Psychiatry, Central Institute of Mental Health (CIMH), Medical Faculty Mannheim Heidelberg University Mannheim Germany; ^20^ ZIPP Biobank, Central Institute of Mental Health (CIMH), Medical Faculty Mannheim Heidelberg University Mannheim Germany; ^21^ Department of Psychiatry and Neurosciences (CCM) Charité – Universitätsmedizin Berlin, corporate member of Freie Universität Berlin and Humboldt‐Universität zu Berlin Berlin Germany

**Keywords:** alcohol craving, ecological momentary assessment, EMA, gestagen, loss of control, sex hormones

## Abstract

Alcohol use disorder (AUD) causes a high burden worldwide. The menstrual cycle and the progesterone‐to‐estradiol ratio are related to alcohol consumption in females and males with AUD. However, the underlying mechanisms triggering problem drinking in everyday life remain to be determined. This longitudinal multicentre study analysed associations of menstrual cycle phases (1316 of 70 naturally cycling females with AUD) and progesterone‐to‐estradiol ratios (quantified in 567 blood samples of 256 males with AUD) with real‐world 12‐month data on loss of control over alcohol consumption and craving measured via 17296 and 10482 smartphone entries using general linear mixed models. Females with severe AUD stated less frequent loss of control in the midluteal and late luteal phases characterized by high progesterone‐to‐estradiol ratios compared to the follicular phase. In the total female sample, craving was lower during the late luteal and menstrual phases compared to the follicular phase. Similarly, males with moderate and severe AUD reported less frequent loss of control in phases of a high progesterone‐to‐estradiol ratio. The identified biobehavioural interplay suggests that increasing progesterone relative to estradiol may assist individuals with AUD in re‐establishing control over alcohol consumption and reducing craving. When employed in conjunction with customized just‐in‐time adaptive digital interventions, this approach has the potential to evolve into a novel precision medicine strategy for AUD. The findings also underscore the necessity for the development of treatments tailored to the phases of the menstrual cycle, thereby addressing the critical underrepresentation of females in AUD research.

## Graphical abstract

Alcohol use disorder (AUD) is a major global health burden. The study analysed female sex hormones on drinking outcomes in a multicentre longitudinal setting involving daily EMA in 70 women and 256 men over 12 months. Women reported fewer craving and loss of control during the mid‐ and late luteal cycle phases. Higher progesterone‐to‐estradiol ratios were linked to less craving and loss of control in males with AUD. Hormone‐ and cycle phase‐tailored digital interventions could offer a new personalized treatment approach. Further research should address the underrepresentation of women in AUD studies.

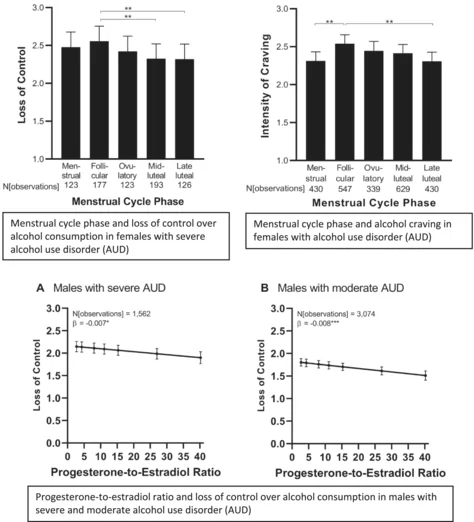

## 1 Introduction

Alcohol use disorder (AUD) is a devastating public health problem. With 46 million women and 237 million men affected worldwide, AUD is very widespread (2% of women and 9% of men) and responsible for 145 000 deaths per year (0.3% of all deaths) [1, 2]. Moreover, AUD causes annually 18.5 million disability‐adjusted life years (DALYs), which represent 0.7% of all DALYs [1], and is associated with a high socioeconomic burden [3]. To develop novel effective and safe digital treatments a better understanding of the mechanisms underlying AUD is crucial.

Despite the considerable prevalence of AUD in women, research over the past decades has notably overlooked the examination of problem drinking in females. (We here refer to sex rather than gender and thus use the terms female and male instead of woman and man.) A recent study [4], focusing on females with AUD, revealed that phases of the menstrual cycle significantly influence the amount of alcohol consumed. Drinking behaviour is at its lowest during the late luteal phase, characterized by a high progesterone‐to‐estradiol ratio, and peaks during the follicular and ovulatory phases, which are characterized by low progesterone‐to‐estradiol ratios; in support of this, a higher progesterone‐to‐estradiol ratio is related to reduced alcohol drinking in males with AUD [4]. Accordingly, previous studies support a role for progesterone and estradiol serum concentrations in AUD, with distinct effects observed in both females and males [5, 6]. To translate these finding into digital treatments, more precise knowledge of the effects of the menstrual cycle phase and in particular the progesterone‐to‐estradiol ratio [7] on behaviours central to addiction is required. Moreover, research needs to identify real‐life targets for digital interventions [8] based on the dynamical systems view [9] beyond merely epidemiological cross‐sectional associations. In the context of the effects of menstrual cycle and particularly the progesterone‐to‐estradiol ratio on problem drinking, the loss of control over alcohol consumption and the craving for alcohol may emerge as relevant and accessible treatment targets.

In healthy females, the inhibition of prepotent responses in a stop‐signal task was found to be more efficient [10] and craving for alcohol less intense [11] during the luteal phase in comparison to the follicular phase. A higher progesterone‐to‐estradiol ratio was related to a longer time to smoking in a smoking resistance task in female daily smokers [12]. Also, higher serum progesterone concentrations were associated with a stronger luteal frontal–striatal activation in naturally cycling females [13], lower craving in postmenopausal females with AUD [6], a blunted craving response to social stress in females with cocaine use disorder [14] and lower anxiety and craving following administration of yohimbine, a pharmacological stressor, in cocaine‐dependent females [15]. However, these cross‐sectional and laboratory studies are limited in sample size, and the translation into daily life to enable effective digital interventions has yet to be demonstrated.

### 1.1 Aims of the Study and Hypotheses

To identify real‐life behavioural mechanisms, we here studied associations of the menstrual cycle phase (as an indicator of changes in the progesterone‐to‐estradiol ratio) in females with AUD and the progesterone‐to‐estradiol ratio in males with AUD with smartphone‐based ecological momentary assessments on loss of control over alcohol consumption and alcohol craving. Although the influence of progesterone and estradiol has been mostly studied in females with AUD (due to the natural cyclic variation), females are underrepresented in AUD research in general; to address this critical research gap, we here followed a rigorous sex‐segregated approach.

We expected interaction effects of menstrual cycle phase and progesterone‐to‐estradiol ratio with AUD severity, which is a relevant moderator in similar studies and linked to loss of control and craving [16], and tested the following hypotheses using 12‐month data from smartphone entries of a longitudinal multicentre study:

*Females with AUD:* Depending on AUD severity, the frequency of loss of control over consumption and the intensity of craving vary across the menstrual cycle phases with minima during the mid−/late luteal phases and maxima during the follicular/ovulatory phases. The progesterone‐to‐estradiol ratio peaks during the mid−/late luteal phases and reaches its nadir during the follicular/ovulatory phases.
*Males with AUD:* Higher progesterone‐to‐estradiol ratios are directly related to less frequent loss of control over consumption and less intense alcohol craving, and these associations are moderated by AUD severity.

## 2 Methods

### 2.1 Study Design

This longitudinal multicentre study is part of the German Collaborative Research Center Transregio 265 (TRR 265) project (Study registration: DRKS00020580) [17]. The design involves individual real‐world smartphone entries on loss of control over alcohol consumption and alcohol craving over a 12‐month period and four onsite study visits with blood drawings (baseline and after 4, 8 and 12 months). For further details on the study design, see Figure S1. The study was approved by the local ethics committees of the study sites. The participants provided written informed consent. We involved people with related lived experience in design of the study and interpretation of the data.

Out of 3492 individuals screened, 672 females and males (self‐reported sex) were enrolled in Berlin (Charité–Universitätsmedizin Berlin), Dresden (Technische Universität Dresden) and Mannheim (Central Institute of Mental Health) between February 2020 and July 2022. The project was conducted in Germany. Mainly White participants were included, but we did not ascertain ethnicity. General inclusion and exclusion criteria as well as the additional inclusion criteria for premenopausal naturally cycling females are reported in Supporting Information. After exclusion of participants lacking ecological momentary assessment data for loss of control, craving and alcohol use or without sufficient information on menstrual cycle (females) and progesterone‐to‐estradiol ratios (males), we analysed data from 70 premenopausal naturally cycling females and 256 males with AUD.

### 2.2 Menstrual Cycle Phases

Menstrual cycle information was assessed using the following questions [18, 19]: *During the previous 3 months, did you have a regular cycle? [Yes/No]. How long does your cycle last? [Less than 21 days/21 to 23 days/24 to 26 days/27 to 29 days/30 to 32 days/33 to 35 days/more than 35 days]. When did your last menstruation start? [Day|Month|Year]. Do you take contraceptives or other hormones? [Yes/No]*. To classify the menstrual cycle phase, we used an approach [20], which takes into account that variations in cycle length are primarily due to differences in the length of the follicular phase. The phases of the menstrual cycle are a proxy for the progesterone‐to‐estradiol ratio and facilitate the comparison of results between women and men.

### 2.3 Ecological Momentary Assessment

Participants were asked to provide real‐world smartphone entries (reminding alarm at noon that could be postponed until 8 PM): (1) *During the previous 8 days, it happened that I no longer had any control over my alcohol consumption*. 6‐point Likert scale from 1, *Never* to 6, *Always* (presented every eighth day; we analysed days without alcohol use and no loss of control (=Never) as well as all days with alcohol use); (2) *How much craving for alcohol did you experience on average during the last 24 h (the desire for alcohol during the time when you did not drink)?* 7‐point Likert scale from 1, *No craving* to 7, *Extreme craving* (presented every other day) (for questions in German language see Supporting Information). We reached sound adherence rates (Median): Loss of control—females 77% and males 69%; craving—females 80% and males 80%. For details on the assessment of alcohol consumption, see [4, 16]. As described previously [4], we analysed ecological momentary assessment data from 56 days after baseline, 28 days before and 28 days after Follow‐ups 1 and 2 and 56 days before Follow‐up 3 (Figure S1).

### 2.4 Measurement of Blood Hormone Concentrations

After blood drawing during the four study visits, the serum vials were left at room temperature for 0.5 to 2 h and then centrifuged for 12 min at 1100 g and 4°C. Serum aliquots were stored at −80°C at the Biobank of Psychiatric Diseases Mannheim. For hormone quantifications, we used the Progesterone ELISA (RE52231; IBL International GmbH, Hamburg, Germany; duplicates of 25‐μL sample in parallel to standard curves from 0.15 to 40 ng/mL; intra‐ and interassay coefficients of 3.0% and 10.2%) and the Estradiol ELISA (EIA‐2693; DRG Instruments GmbH, Marburg, Germany; duplicates of 25‐μL sample in parallel to standard curves from 12.5 to 2000 pg/mL; intra‐ and interassay coefficients of 7.0% and 12.9%). The progesterone‐to‐estradiol ratio was calculated as (progesterone [ng/mL] * 1000 [pg/ng])/(estradiol [pg/mL]).

### 2.5 Statistical Analysis

We used general linear mixed models to investigate the AUD severity‐specific associations of menstrual cycle phase in females and progesterone‐to‐estradiol ratio in males (predictors) with the outcomes loss of control over alcohol consumption and intensity of alcohol craving (dependent variables). The multilevel models included main effects of menstrual cycle phase in females and progesterone‐to‐estradiol ratio in males, the AUD severity (severe vs. moderate vs. mild) and the interaction effect between menstrual cycle phase in females or progesterone‐to‐estradiol ratio in males and AUD severity. In case of no indication for a relevant interaction effect, main effects of menstrual cycle phase, or progesterone‐to‐estradiol ratio, and AUD severity were tested. We report *F*‐tests for all interaction and main effects, parameter estimates *β*, and *t*‐values for *β* when predictors had ≥ 3 categories. The models were adjusted for weekend days (vs. weekdays) and COVID‐19 lockdown phase (vs. no lockdown phase) due to their known impact on alcohol use [16] as well as time of blood collection in males to account for circadian hormonal variation.

The study design of only four blood drawings during the 12‐month follow‐up did not permit to directly test the association between progesterone‐to‐estradiol ratio and behavioural outcomes in the female subsample. We thus used the menstrual cycle phase as an indicator of the hormone ratio. To validate menstrual cycle phase for this purpose, we computed a general linear mixed model with menstrual cycle phase as predictor and progesterone‐to‐estradiol ratio as dependent variable. This model was adjusted for time of blood collection to account for circadian rhythm.

Random intercepts were included in the mixed models to account for the nested data structure within participants (Level 1: smartphone entries or progesterone‐to‐estradiol ratio; Level 2: participants). We did not include the phase as an additional level in the random effects, because we did not expect a benefit for modelling one more individual level in addition to the random intercept for person. As a covariance structure, we used variance components, and for the computation of marginal means, we used observed margins as weighting scheme. We used SPSS for Windows, version 29.0; SAS, version 9.4; and GraphPad Prism 8. The significance level was set to *α* = 0.05 (two‐sided).

## 3 Results

### 3.1 Sample Descriptions

In total, we analysed 17 296 loss of control and 10 482 craving smartphone‐based real‐world observations as well as data of 1316 menstrual cycle phases and hormone parameters of 636 blood samples collected from 70 females and 256 males with AUD (for further details, see Table 1).

**TABLE 1:** Sample descriptions of premenopausal naturally cycling females and males with alcohol use disorder (AUD).

	Females with AUD			Males with AUD		
	*N*	M or %	SD	*N*	M or %	SD
**Sample characteristics**						
*N* (cases)	70			256		
Age (years)		29.8	6.9		38.0	12.7
Marital status						
% Single	46	66%		114	46%	
% Living in marriage or partnership	19	27%		111	45%	
% Living separately	2	3%		10	4%	
% Divorced	2	3%		13	5%	
% Widowed	1	1%		1	0%	
% Employed last 3 months	61	87%		201	81%	
Highest school qualification						
% Pupil at a general education school/in training	2	3%		8	3%	
% Secondary general school certificate^1^	0	0%		5	2%	
% General Certificate of Secondary Education^2, 3^	9	13%		40	16%	
% Advanced technical college certificate^4^	8	11%		27	11%	
% General Certificate of Education^5^	50	71%		165	66%	
% Another school degree	1	1%		4	2%	
Number of fulfilled AUD criteria		4.3	1.6		4.0	1.6
% mild AUD (2–3 criteria met)	26	37%		114	45%	
% moderate AUD (4–5 criteria met)	27	39%		93	36%	
% severe AUD (≥ 6 criteria met)	17	24%		49	19%	
% 3‐month prevalence tobacco smoking	44	63%		138	54%	
% 3‐month prevalence cannabis use	26	37%		71	28%	
**Real‐world e‐diary entries (EMA)**						
*N* (cases)	70			256		
*N* (menstrual cycle phases)	1316					
*N* (observations) loss of control	3787			13 509		
*N* (observations) craving	2375			8107		
Daily alcohol use (g/day)		24.9	15.4		40.6	27.5
Alcohol craving		2.3	0.8		2.3	1.0
Loss of control		1.9	0.9		1.8	0.9
**Blood samples and hormone measures**						
*N* (cases)	46			256		
*N* (observations)	69			567		
Progesterone‐to‐estradiol ratio		46.0^6^	62.5		12.6	7.5

*Note:* The table reports absolute frequencies, mean values (M) or relative frequencies and standard deviations (SD). Progesterone‐to‐estradiol ratio, [progesterone (ng/mL) * 1,000 (pg/ng)] / [estradiol (pg/mL)] ratio. ^1^Corresponds to ‘Hauptschulabschluss’, ^2^to ‘Realschulabschluss’, ^3^to ‘Abschluss polytechnische Oberschule’, ^4^to ‘Fachhochschulreife’ and ^5^to ‘Abitur’ in the German educational system. ^6^For the progesterone‐to‐estradiol ratios specific to the menstrual cycle phases, see Figure S2.

Abbreviations: AUD, alcohol use disorder; EMA, ecological momentary assessment.

### 3.2 Premenopausal Naturally Cycling Females With Alcohol Use Disorder

In females, the menstrual cycle phase‐by‐AUD severity interaction was related to loss of control over alcohol consumption (Table S1). The AUD severity‐specific *F*‐tests (simple effects) and post hoc comparisons (*t*‐tests) showed an association in females with severe AUD (Figure 1; *F*(4, 3713) = 3.010, *p* = 0.017): Loss of control was less frequent during the mid‐ and late luteal phases in comparison to the follicular phase (mean differences [95% CIs] −0.230 [−0.382, −0.079], *t*(3715) = −2.987, *p* = 0.003 and −0.238 [−0.408, −0.068], *t*(3713) = −2.736, *p* = 0.006); there were no such significant effects in females with mild or moderate AUD (*F*(4, 3707) = 1.921, *p* = 0.104; *F*(4, 3714) = 1.680, *p* = 0.152). Loss of control over alcohol consumption was more frequent on weekend days compared to weekdays (Table S1).

**FIGURE 1:**
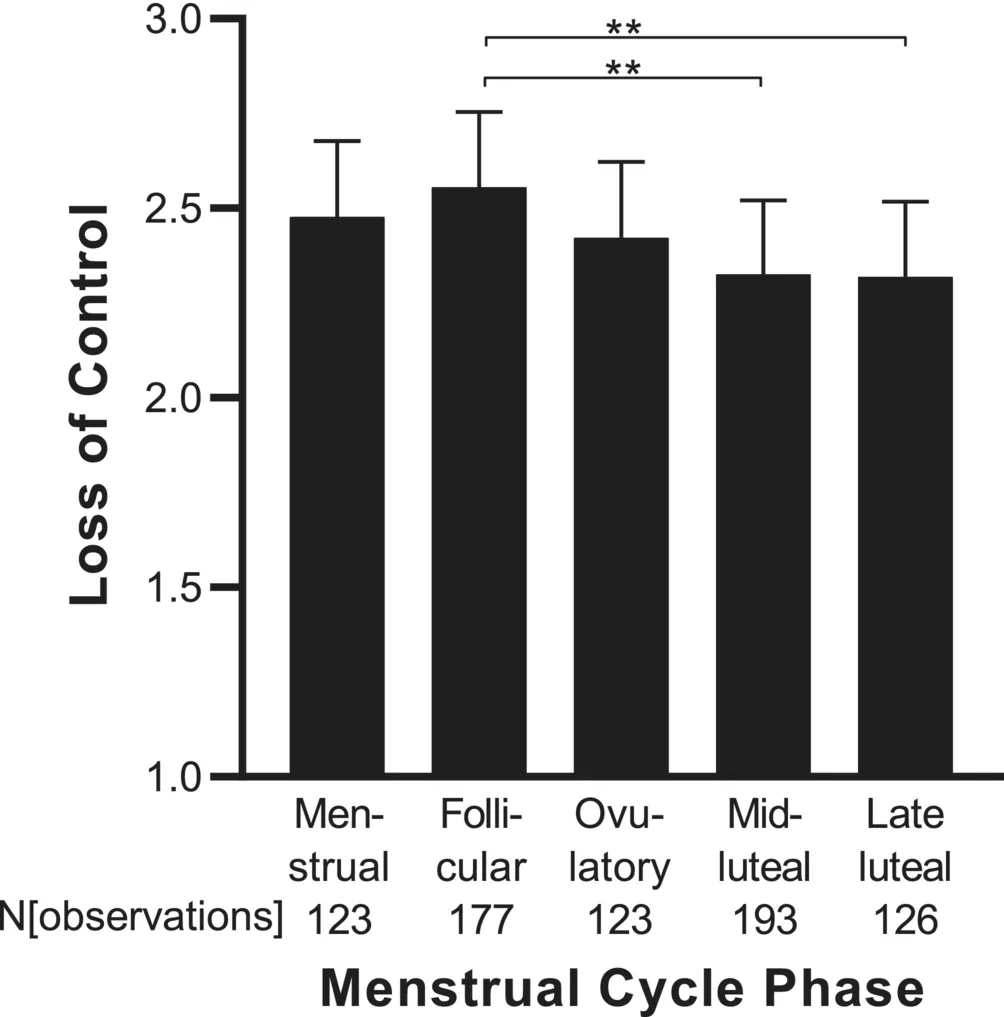
Menstrual cycle phase and loss of control over alcohol consumption in females with severe alcohol use disorder (AUD). The figure illustrates the significant menstrual cycle phase effect on loss of control over alcohol consumption in females with severe AUD (Likert Scale 1 to 6 from *Never* to *Always*; for details of the overall statistical model, see Table S1). The graph shows marginal predicted means and standard errors. **p < 0.01.

The menstrual cycle phase‐by‐AUD severity interaction was not significantly associated with alcohol craving (Table S2). Subsequently, we found a main effect of menstrual cycle phase on craving with lower intensity during the late luteal and menstrual phases in comparison to the follicular phase (mean differences −0.232 [−0.374, −0.091], *t*(2313) = −3.222, *p* = 0.001 and −0.226 [−0.367, −0.085], *t*(2309) = −3.139, *p* = 0.002; Figure 2). Craving was higher on weekend days than on weekdays (Table S2).

**FIGURE 2:**
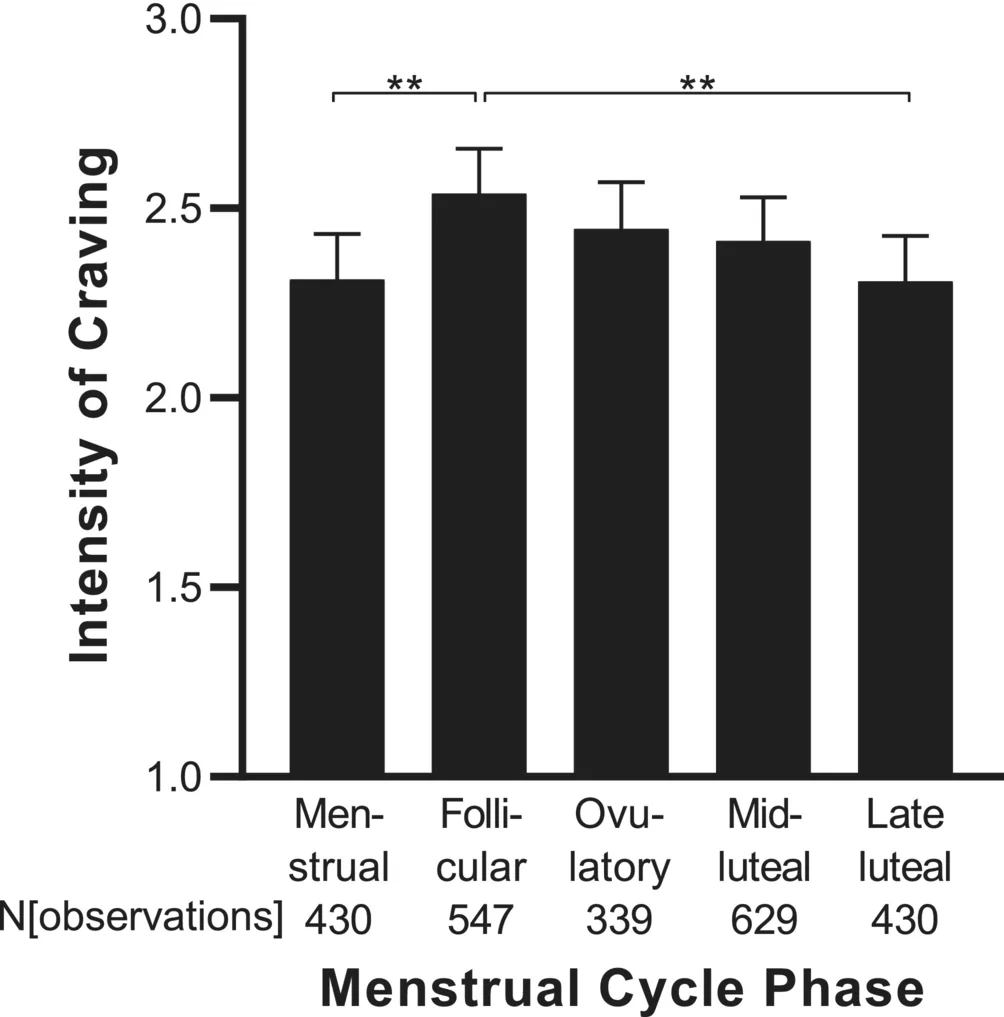
Menstrual cycle phase and alcohol craving in females with alcohol use disorder (AUD). The figure illustrates the significant change of intensity of alcohol craving across the menstrual cycle in premenopausal naturally cycling females with AUD (Likert Scale 1 to 7 from *No* *craving* to *Extreme craving*; for details of the overall statistical model, see Table S2). The graph shows marginal predicted means and standard errors. **p < 0.01.

The changes in loss of control over alcohol consumption and alcohol craving across the menstrual cycle were paralleled by variations in progesterone‐to‐estradiol ratio. The analyses indicated peak progesterone‐to‐estradiol ratios during the mid‐ and late luteal phases and minima during the follicular and ovulatory phases, which supports the validity of the menstrual cycle phase as a proxy for longitudinal changes in the progesterone‐to‐estradiol ratio (Figure S1, Table S3).

### 3.3 Males With Alcohol Use Disorder

In males, the progesterone‐to‐estradiol ratio‐by‐AUD severity interaction was related to loss of control over alcohol consumption (Table S4). A higher progesterone‐to‐estradiol ratio was associated with less frequent loss of control in the groups of males with severe and moderate AUD (Figure 3; severity‐specific *t*‐tests (simple slopes): *β* = −0.007 [−0.012, −0.001], *t*(13257) = −2.51, *p* = 0.012 and *β* = −0.008 [−0.012, −0.003], *t*(13460) = −3.40, *p* < 0.001); there were no such significant effects in males with mild AUD (*β* = 0.003, t(13402) = 1.28, *p* = 0.200). Weekend days (vs. week days) were associated with more frequent loss of control over alcohol consumption (Table S4). Neither the interaction effect of progesterone‐to‐estradiol ratio‐by‐AUD severity nor the main effect of the progesterone‐to‐estradiol ratio on intensity of alcohol craving was significant (Table S5).

**FIGURE 3:**
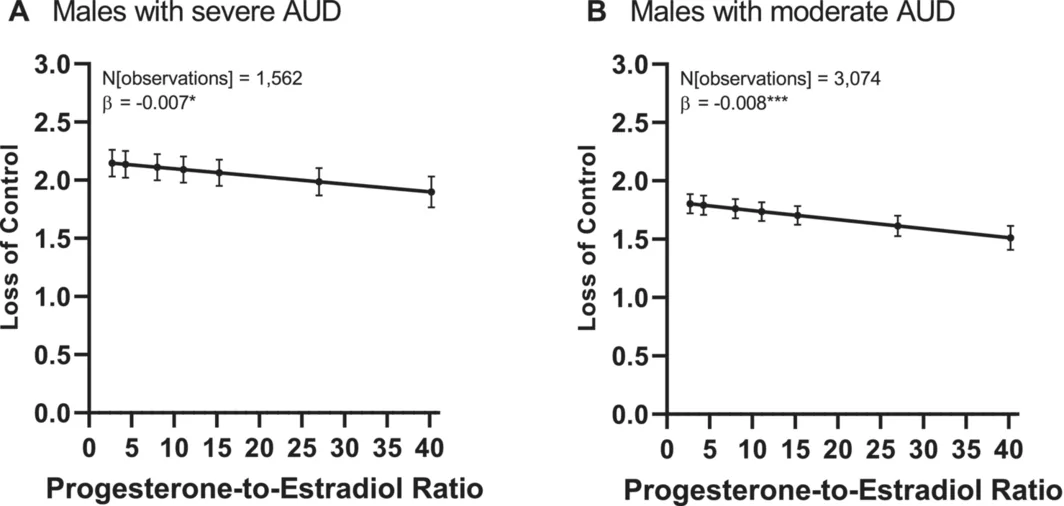
Progesterone‐to‐estradiol ratio and loss of control over alcohol consumption in males with severe and moderate alcohol use disorder (AUD) The figure illustrates the significant progesterone‐to‐estradiol ratio effect on loss of control over alcohol consumption in males with severe (**A**) and moderate (**B**) AUD (Likert Scale 1 to 6 from *Never* to *Always*; for details of the overall statistical model, see Table S4). The graph shows marginal predicted means and standard errors at 1, 5, 25, 50, 75, 95 and 99 percentiles of the progesterone‐to‐estradiol ratios. Progesterone‐to‐estradiol ratio, [progesterone (ng/mL) *1000 (pg/ng)]/[estradiol (pg/mL)] ratio. *p < 0.05, ***p < 0.001.

## 4 Discussion

The results give novel real‐life insights into the biobehavioural dynamical mechanisms underlying AUD in females and provide converging evidence in males. The study demonstrates that in the everyday life of females with AUD loss of control over alcohol consumption and craving for it are reduced in the luteal phase of the menstrual cycle. Therefore, this study does not only agree with the earlier observations from cross‐sectional and laboratory investigations of higher inhibitory control [10], lower craving for alcohol [11] and less social drinking [21] during the luteal phase compared to the follicular phase in healthy females; the findings also extend the previously reported nadir in daily alcohol consumption during the late luteal phase [4] to real‐life behavioural triggers assessed via innovative digital technology.

This study revealed that the fluctuations in loss of control over alcohol consumption and craving across the menstrual cycle correspond to changes in the progesterone‐to‐estradiol ratio. Therefore, the findings connect higher progesterone relative to estradiol activities with less frequent loss of control and less intense craving. This assumption is supported by previous studies showing that higher blood progesterone (relative to estradiol) activities are associated with a longer time to smoking in a smoking resistance task in female daily smokers [12], higher luteal frontal–striatal activation in naturally cycling females [13], lower alcohol craving in postmenopausal female inpatients with alcohol dependence [6] and attenuated reactivity in craving and anxiety to social and pharmacological stressors in females with cocaine use disorder [14, 15].

The AUD severity moderated the association between menstrual cycle phase and loss of control over consumption, whereas the menstrual cycle phase‐by‐AUD severity interaction was not significantly related to craving. This observation is consistent with results indicating that neural circuits underlying the conscious craving differ from those of the more automatized loss of control [22]. Hence, regional variations in brain function between AUD severities might account for the observation of a menstrual cycle‐by‐AUD severity interaction effect on loss of control in parallel to a main effect of menstrual cycle on craving.

In males with severe and moderate AUD, a higher blood progesterone‐to‐estradiol ratio was directly associated with less frequent loss of control over consumption. This aligns with the previous finding of less daily alcohol consumption during phases with higher blood progesterone‐to‐estradiol ratios in males with AUD [4].

The study results suggest that increasing progesterone relative to estradiol may serve as a novel treatment target for AUD. Indeed, experimental studies suggest that the administration of exogenous progesterone may improve phenotypes of cocaine and tobacco use disorders [23, 24]. However, to the best of our knowledge, there is a lack of studies regarding the effects of progesterone administration on alcohol consumption and related phenotypes in patients with AUD. Nonetheless, reviews suggest that progestogen‐related neurosteroids are involved in the comorbidity of post‐traumatic stress disorder and AUD [25, 26]. Moreover, a placebo‐controlled laboratory study including 12 males and one female with both post‐traumatic stress disorder and AUD revealed that progesterone administration over 3 days can reduce craving elicited by exposure to trauma scripts [27]. The sex‐segregated results of the present study underscore the role of the menstrual cycle and the progesterone‐to‐estradiol ratio in loss of control over alcohol consumption and intensity of alcohol craving in individuals with AUD. Particularly in those with severe AUD increasing progesterone relative to estradiol concentrations might be beneficial. The clinical importance of these results is underscored by our previous finding that the late luteal phase (marked by a high progesterone‐to‐estradiol ratio) in females with AUD and a high progesterone‐to‐estradiol ratio in males with AUD are associated with a higher probability of binge drinking days and a higher amount of daily alcohol consumption [4]. Thus, treatment with progesterone might evolve as a strategy to support regaining control over alcohol consumption, reduce alcohol craving and ultimately attenuate problem drinking in both females and males with AUD.

Beyond the pure pharmacological view, the study results also indicate that increasing progesterone relative to estradiol may enhance behavioural treatments aimed at protecting against momentary loss of control over alcohol consumption and craving, features that are especially pertinent to just‐in‐time adaptive interventions [28]. In general, the study results also encourage the progression of therapeutic approaches tailored to menstrual cycle phases in females with AUD [4, 29].

### 4.1 Strengths and Limitations

The observational design of this study does not permit conclusions about the directionality and causality of the associations between hormonal parameters and the here investigated drinking behaviours. For example, the hormonal variations may influence craving and loss of control, alcohol consumption may alter sex hormone concentrations vice versa, and unmeasured confounders (e.g., stress) may affect both. To this end, future experimental setups modulating progesterone and/or estradiol activities are needed, for example, through exogenous progesterone administration or transient pharmacological suppression of the oestrogen signalling pathway. However, the approach segregating by sex, coupled with a large sample size of individuals with AUD and the uniquely large ecological momentary assessment dataset, ensures reproducibility and generalizability. A particular focus was given to females diagnosed with AUD, with strictly adherence to the Sex and Gender Equity in Research Guidelines (SAGER) [4, 30]. Ecological momentary assessment technology enables real‐world insights into distinct environmental contexts, reduces recall bias and facilitates access to within‐person variability, thus enabling future‐oriented digital phenotyping in clinical research and integrating the dynamical systems view [8, 9]. In order to translate these findings into personalized and effective digital treatments, future clinical trials are needed. These trials should combine experimental manipulations at both the pharmacological and behavioural levels within everyday life, thereby establishing causal relationships.

### 4.2 Conclusion and Outlook

Using innovative long‐term ecological momentary assessment combined with repeated laboratory blood sampling in a large cohort of females and males with AUD, we identify the menstrual cycle and the progesterone‐to‐estradiol ratio to be modulators of loss of control over alcohol consumption and craving for alcohol in everyday life. The observed interplay between biological and behavioural mechanisms in day‐to‐day life suggests that the integration of progesterone administration with innovative, just‐in‐time adaptive digital interventions via smartphones may evolve into a novel precision medicine strategy in females and males with AUD derived from an innovative combination of biological and digital in‐depth phenotyping [31]. Additionally, the findings suggest that cycle phase‐tailored therapeutic approaches are a promising direction for AUD treatment, thus addressing the critical underrepresentation of females in AUD research [32].

## Author Contributions

**Hoffmann, Reinhard, Mühle, Bach, D. Reichert, Boroumand‐Jazi, Nayman, Sommer, Spanagel, Banaschewski, Deserno, Ebner‐Priemer, Meyer‐Lindenberg, Rapp, Witt, Smolka, Heinz, Tost, Kiefer, M. Reichert, Lenz:** study concept and design. **All authors:** acquisition, analysis or interpretation of data. **Hoffmann, Reinhard, M. Reichert, Lenz:** drafting of the manuscript. **All authors:** critical revision of the manuscript for important intellectual content. **Hoffmann, Reinhard:** statistical analysis. **Beck, Müller, Sommer, Spanagel, Banaschewski, Deserno, Ebner‐Priemer, Meyer‐Lindenberg, Rapp, Witt, Smolka, Heinz, Tost, Kiefer, M. Reichert, Lenz:** obtained funding. **Mühle, Kornhuber, Banaschewski, Deserno, Ebner‐Priemer, Meyer‐Lindenberg, Rapp, Witt, Smolka, Heinz, Tost, Kiefer, M. Reichert, Lenz:** administrative, technical and/or material support. **Bach, Banaschewski, Deserno, Ebner‐Priemer, Rapp, Witt, Smolka, Heinz, Tost, Kiefer, M. Reichert, Lenz:** study supervision.

## Funding

This work was supported by the Deutsche Forschungsgemeinschaft (402170461).

## Disclosure

Tobias Banaschewski served in an advisory or consultancy role for eye level, Infectopharm, Lundbeck, Medice, Neurim Pharmaceuticals, Oberberg GmbH, Roche and Takeda. He received conference support or speaker's fee from Janssen, Medice and Takeda. He received royalties from Hogrefe, Kohlhammer, CIP Medien, Oxford University Press; the present work is unrelated to these relationships. Ulrich W. Ebner‐Priemer is a consultant for Boehringer Ingelheim. Andreas S. Meyer‐Lindenberg has received consultant fees from Daimler und Benz Stiftung, EPFL Brain Mind Institute, Fondation FondaMental, Hector Stiftung II, Invisio, Janssen‐Cilag GmbH, Lundbeck A/S, Lundbeckfonden, Lundbeck Int. Neuroscience Foundation, Neurotorium, MedinCell, The LOOP Zürich, University Medical Center Utrecht, University of Washington, Verein für Mentales Wohlbefinden, von Behring‐Röntgen‐Stiftung; speaker fees from Ärztekammer Nordrhein, Caritas, Clarivate, Dt. Gesellschaft für Neurowissenschaftliche Begutachtung, Gentner Verlag, Landesärztekammer Baden‐Württemberg, LWL Bochum, Northwell Health, Ruhr University Bochum, Penn State University, Society of Biological Psychiatry, University Prague, Vitos Klinik Rheingau and editorial and/or author fees from American Association for the Advancement of Science, ECNP, Servier Int., Thieme Verlag.

## Conflicts of Interest

The authors declare no conflicts of interest.

## Supporting information


**Figure S1:** Study design.
**Figure S2:** Menstrual cycle phase and progesterone‐to‐estradiol ratio in females with alcohol use disorder (AUD).
**Table S1:** Loss of control over alcohol consumption in naturally cycling females with alcohol use disorder (AUD): AUD severity‐specific association with the menstrual cycle phase (interaction effects model).
**Table S2:** Intensity of alcohol craving in naturally cycling females with alcohol use disorder (AUD): AUD severity‐specific association with menstrual cycle phase (interaction effects model) and overall association with menstrual cycle phase (main effects model).
**Table S3:** Progesterone‐to‐estradiol ratio in naturally cycling females with alcohol use disorder (AUD): association with menstrual cycle phases.
**Table S4:** Loss of control over alcohol consumption in males with alcohol use disorder (AUD): AUD severity‐specific association with the progesterone‐to‐estradiol ratio.
**Table S5:** Intensity of alcohol craving in males with alcohol use disorder (AUD): AUD severity‐specific association with the progesterone‐to‐estradiol ratio (interaction effects model) and overall association with the progesterone‐to‐estradiol ratio (main effects model).

## Data Availability

The protocol, in addition to the methodological and statistical details, can be found in the Supporting Information. Individual participant data can be requested from the authors in an anonymized form.
