# Supplementary material for: Menstrual Cycle and Progesterone‐to‐Estradiol Ratio Associated With Loss of Control and Craving in Alcohol Use Disorder: A Sex‐Segregating Longitudinal Study
Source: Addict Biol. 2026 Oct 4;31(10):e70192. doi: 10.1111/adb.70192 (PMC13635626; doi:10.1111/adb.70192)
Supplement: Supplementary file 1 [file ADB-31-e70192-s001.docx]

Data supplement for Hoffmann et al., Menstrual cycle and progesterone-to-estradiol ratio associated with loss of control and craving in alcohol use disorder: a sex-segregating longitudinal study

**CONTENTS**

[**Supplementary Methods**	2](#_Toc186022380)

[Study design	2](#_Toc186022381)

[Classification of menstrual cycle phases	2](#_Toc186022382)

[Ecological momentary assessment	2](#_Toc186022383)

[Definitions	2](#_Toc186022384)

[**Supplementary Figure 1.** Study design	4](#_Toc186022385)

[**Supplementary Figure 2.** Menstrual cycle phase and progesterone-to-estradiol ratio in females with alcohol use disorder (AUD)	5](#_Toc186022386)

[**Supplementary Table 1.** Loss of control over alcohol consumption in naturally cycling females with alcohol use disorder (AUD): AUD severity-specific association with the menstrual cycle phase (interaction effects model)	6](#_Toc186022387)

[**Supplementary Table 2.** Intensity of alcohol craving in naturally cycling females with alcohol use disorder (AUD): AUD severity-specific association with menstrual cycle phase (interaction effects model) and overall association with menstrual cycle phase (main effects model)	7](#_Toc186022388)

[**Supplementary Table 3.** Progesterone-to-estradiol ratio in naturally cycling females with alcohol use disorder (AUD): association with menstrual cycle phases	8](#_Toc186022389)

[**Supplementary Table 4.** Loss of control over alcohol consumption in males with alcohol use disorder (AUD): AUD severity-specific association with the progesterone-to-estradiol ratio	8](#_Toc186022390)

[**Supplementary Table 5.** Intensity of alcohol craving in males with alcohol use disorder (AUD): AUD severity-specific association with the progesterone-to-estradiol ratio (interaction effects model) and overall association with the progesterone-to-estradiol ratio (main effects model)	9](#_Toc186022391)

[**Non-author collaborators of the ReCode Consortium^5,6^**	10](#_Toc186022392)

[**References**	10](#_Toc186022393)

# **Supplementary Methods**

## Study design

General inclusion criteria were: At least 2 alcohol use disorder (AUD) criteria fulfilled according to the Diagnostic and Statistical Manual of Mental Disorders, Fifth Edition (DSM-5) [1]; aged between 16 and 65 years; capable to understand the study protocol; written informed consent.

General exclusion criteria were: Intake of central nervous system agents in the last 10 days or less than 5 half-life periods ago; contraindications for magnetic resonance tomographic scans; DSM-5 bipolar disorder, psychotic disorder, schizophrenia or disorder from the schizophrenic spectrum; substance addiction except for alcohol, nicotine, and cannabis in medical history; severe head injury or other severe diseases of the central nervous system in medical history; pregnancy, breastfeeding.

Additional inclusion criteria for premenopausal naturally cycling females: Age ≤ 42 years (= 50 years as median age at last menses in German females [2] and 8 years to account for perimenopause [3]); self-report of a regular cycle (previous 3 months); no intake of hormonal contraceptives or other hormones and no use of hormonal intrauterine or vaginal devices, subdermal implants, or contraceptive patches.

## Classification of menstrual cycle phases

Cycle days for menstrual, follicular, ovulatory, mid-luteal, and late luteal phases; cycle length 21-23 days: 1-5, 6, 7-10, 11-17, 18-22; cycle length 24-26 days: 1-5, 6-9, 10-13, 14-20, 21-25; cycle length 27-29 days: 1-5, 6-12, 13-16, 17-23, 24-28; cycle length 30-32 days: 1-5, 6-15, 16-19, 20-26, 27-31; cycle length 33-35 days: 1-5, 6-18, 19-22, 23-29, 30-34.

## Ecological momentary assessment

Participants were asked to provide real-world smartphone entries (reminding alarm at noon that could be postponed until 8 PM):

Loss of control – Question in German language: *Es kam in den vergangenen 8 Tagen vor, dass ich keine Kontrolle mehr über meinen Alkoholkonsum hatte.* 6-Punkte Likert-Skala: 1 = niemals, 6 = immer.

Craving – Question in German language: *Wie viel Verlangen nach Alkohol (der Wunsch nach Alkohol während der Zeit, in der Sie nicht tranken,) haben Sie im Durchschnitt in den letzten 24h empfunden?* 7-Punkte Likert-Skala: 1 = keines, 7 = extremes Verlangen.

## Definitions

COVID-19 lockdown phases: We classified the following days as hard lockdown phases: March 22nd to Mai 5th, 2020 and December 16th, 2020 to February 28th, 2021 [4]

(<https://www.bundesregierung.de/breg-de/themen/coronavirus/besprechung-der-bundeskanzlerin-mit-den-regierungschefinnen-und-regierungschefs-der-laender-vom-22-03-2020-1733248>; <https://www.bundesregierung.de/resource/blob/997532/1827366/69441fb68435a7199b3d3a89bff2c0e6/2020-12-13-beschluss-mpk-data.pdf>; <https://www.bundesregierung.de/breg-de/aktuelles/pressekonferenzen/pressekonferenz-von-bundeskanzlerin-merkel-ministerpraesident-soeder-und-dem-ersten-buergermeister-tschentscher-im-anschluss-an-das-gespraech-mit-den-regierungschefinnen-und-regierungschefs-der-laender-1751050>).

Time of blood collection: Blood drawings took place between 7:40 am and 7:15 pm with a median 3:00 pm (20% in the morning, 80% in the afternoon).

Weekend days and weekdays: Friday to Sunday were classified as weekend days and Monday to Thursday as weekdays.

# **Supplementary Figure 1.** Study design

We followed the naturally cycling females and males with alcohol use disorder (AUD) up to 12 months with continuous ecological momentary assessments (EMA, for details see section Ecological momentary assessment) and onsite study visits including blood drawings at enrollment (study visit 1), 4 months (study visit 2), 8 months (study visit 3), and 12 months (study visit 4). In the female sample, EMA of the following periods were analyzed: 56 days (approximately 2 menstrual cycles) after study visit 1, 28 days before and 28 days after the study visits 2 and 3, and 56 days before study visit 4. In the male sample, the progesterone-to-estradiol ratios quantified in blood drawn during the study visits were interpreted as indicators of the hormone ratios in the 56 days after study visit 1, 28 days before and 28 days after study visits 2 and 3, and 56 days before study visit 4. This approach is identical to that previously described [5].

# **Supplementary Figure 2.** Menstrual cycle phase and progesterone-to-estradiol ratio in females with alcohol use disorder (AUD)

The figure shows that in premenopausal naturally cycling females with AUD the mean progesterone-to-estradiol ratio changes across the menstrual cycle. The graph presents marginal means and standard errors. Progesterone-to-estradiol ratio, [progesterone (ng/mL) * 1,000 (pg/ng)] / [estradiol (pg/mL)] ratio. **p < 0.01, ***p < 0.001. For statistical analysis, see Supplementary Table 3.

| **Supplementary Table 1.** Loss of control over alcohol consumption in naturally cycling females with alcohol use disorder (AUD): AUD severity-specific association with the menstrual cycle phase (interaction effects model) | | | | | | | |
| --- | --- | --- | --- | --- | --- | --- | --- |
|  | β | 95% CI | t | p | F | df1, df2 | p |
| N[cases] = 70  N[observations] = 3,787 |  |  |  |  |  |  |  |
| AUD severity |  |  |  |  | 10.628 | (2, 65) | **< 0.001** |
| Severe | 0.909 | [0.415, 1.403] | 3.661 | **< 0.001** |  |  |  |
| Moderate | 0.089 | [-0.331, 0.509] | 0.422 | 0.422 |  |  |  |
| Mild | [Reference] |  |  |  |  |  |  |
| Weekend days versus weekdays |  |  |  |  | 5.874 | (1, 3705) | **0.015** |
| Weekend days | 0.058 | [0.011, 0.104] |  |  |  |  |  |
| Weekdays | [Reference] |  |  |  |  |  |  |
| No lockdown versus lockdown |  |  |  |  | 2.313 | (1, 3755) | 0.128 |
| No lockdown | 0.088 | [-0.026, 0.202] |  |  |  |  |  |
| Lockdown | [Reference] |  |  |  |  |  |  |
| Cycle phases |  |  |  |  | 1.040 | (4, 3712) | 0.385 |
| Menstrual | 0.002 | [-0.113, 0.116] | 0.027 | 0.978 |  |  |  |
| Follicular | -0.074 | [-0.182, 0.034] | -1.339 | 0.181 |  |  |  |
| Ovulatory | -0.029 | [-0.150, 0.091] | -0.475 | 0.635 |  |  |  |
| Mid-luteal | 0.059 | [-0.045, 0.164] | 1.119 | 0.263 |  |  |  |
| Late luteal | [Reference] |  |  |  |  |  |  |
| Cycle phases-by-AUD severity |  |  |  |  | 3.145 | (8, 3712) | **0.001** |
| Menstrual x severe | 0.158 | [-0.058, 0.373] | 1.436 | 0.151 |  |  |  |
| Menstrual x moderate | -0.118 | [-0.292, 0.055] | -1.341 | 0.180 |  |  |  |
| Menstrual x mild | [Reference] |  |  |  |  |  |  |
| Follicular x severe | 0.312 | [0.110, 0.513] | 3.035 | **0.002** |  |  |  |
| Follicular x moderate | 0.120 | [-0.048, 0.287] | 1.402 | 0.161 |  |  |  |
| Follicular x mild | [Reference] |  |  |  |  |  |  |
| Ovulatory x severe | 0.133 | [-0.085, 0.352] | 1.195 | 0.232 |  |  |  |
| Ovulatory x moderate | -0.012 | [-0.199, 0.174] | -0.131 | 0.896 |  |  |  |
| Ovulatory x mild | [Reference] |  |  |  |  |  |  |
| Mid-luteal x severe | -0.052 | [-0.247, 0.143] | -0.520 | 0.603 |  |  |  |
| Mid-luteal x moderate | -0.101 | [-0.261, 0.060] | -1.231 | 0.218 |  |  |  |
| Mid-luteal x mild | [Reference] |  |  |  |  |  |  |
| Late luteal | [Reference] |  |  |  |  |  |  |
| The multilevel models include fixed and random intercepts. 95% CI, 95% confidence interval; β, regression coefficient. p < 0.05 in bold | | | | | | | |

| **Supplementary Table 2.** Intensity of alcohol craving in naturally cycling females with alcohol use disorder (AUD): AUD severity-specific association with menstrual cycle phase (interaction effects model) and overall association with menstrual cycle phase (main effects model) | | | | | | | |
| --- | --- | --- | --- | --- | --- | --- | --- |
|  | β | 95% CI | t | p | F | df1, df2 | p |
| N[cases] = 70  N[observations] = 2,375 |  |  |  |  |  |  |  |
| AUD severity |  |  |  |  | 1.795 | (2, 65) | 0.174 |
| Severe | 0.467 | [-0.091, 1.025] | 1.662 | 0.100 |  |  |  |
| Moderate | 0.167 | [-0.315, 0.649] | 0.688 | 0.493 |  |  |  |
| Mild | [Reference] |  |  |  |  |  |  |
| Weekend days versus weekdays |  |  |  |  | 16.709 | (1, 2295) | **< 0.001** |
| Weekend days | 0.189 | [0.098, 0.279] |  |  |  |  |  |
| Weekdays | [Reference] |  |  |  |  |  |  |
| No lockdown versus lockdown |  |  |  |  | 3.556 | (1, 2343) | 0.059 |
| No lockdown | 0.054 | [-0.437, 0.009] |  |  |  |  |  |
| Lockdown | [Reference] |  |  |  |  |  |  |
| Cycle phases |  |  |  |  | 4.557 | (4, 2300) | **0.001** |
| Menstrual | 0.054 | [-0.171, 0.279] | 0.472 | 0.637 |  |  |  |
| Follicular | 0.186 | [-0.022, 0.395] | 1.754 | 0.080 |  |  |  |
| Ovulatory | 0.172 | [-0.063, 0.406] | 1.437 | 0.151 |  |  |  |
| Mid-luteal | 0.124 | [-0.078, 0.326] | 1.207 | 0.228 |  |  |  |
| Late luteal | [Reference] |  |  |  |  |  |  |
| Cycle phases-by-AUD severity |  |  |  |  | 1.008 | (8, 2300) | 0.428 |
| Menstrual x severe | -0.159 | [-0.548, 0.230] | -0.803 | 0.422 |  |  |  |
| Menstrual x moderate | -0.038 | [-0.376, 0.300] | -0.219 | 0.827 |  |  |  |
| Menstrual x mild | [Reference] |  |  |  |  |  |  |
| Follicular x severe | 0.254 | [-0.118, 0.626] | 1.338 | 0.181 |  |  |  |
| Follicular x moderate | -0.018 | [-0.340, 0.301] | -0.110 | 0.912 |  |  |  |
| Follicular x mild | [Reference] |  |  |  |  |  |  |
| Ovulatory x severe | 0.041 | [-0.370, 0.452] | 0.195 | 0.845 |  |  |  |
| Ovulatory x moderate | -0.127 | [-0.487, 0.232] | -0.695 | 0.487 |  |  |  |
| Ovulatory x mild | [Reference] |  |  |  |  |  |  |
| Mid-luteal x severe | -0.096 | [-0.448, 0.256] | -0.537 | 0.592 |  |  |  |
| Mid-luteal x moderate | 0.012 | [-0.299, 0.323] | 0.076 | 0.939 |  |  |  |
| Mid-luteal x mild | [Reference] |  |  |  |  |  |  |
| Late luteal | [Reference] |  |  |  |  |  |  |
| AUD severity |  |  |  |  | 1.724 | (2, 65) | 0.186 |
| Severe | 0.468 | [-0.038, 0.974] | 1.847 | 0.069 |  |  |  |
| Moderate | 0.142 | [-0.293, 0.577] | 0.652 | 0.517 |  |  |  |
| Mild | [Reference] |  |  |  |  |  |  |
| Weekend days versus weekdays |  |  |  |  | 17.041 | (1, 2303) | **< 0.001** |
| Weekend days | 0.190 | [0.100, 0.281] |  |  |  |  |  |
| Weekdays | [Reference] |  |  |  |  |  |  |
| No lockdown versus lockdown |  |  |  |  | 3.510 | (1, 2351) | 0.061 |
| No lockdown | -0.213 | [-0.436, 0.010] |  |  |  |  |  |
| Lockdown | [Reference] |  |  |  |  |  |  |
| Cycle phases |  |  |  |  | 3.644 | (4, 2308) | **0.006** |
| Menstrual | 0.006 | [-0.142, 0.155] | 0.084 | 0.933 |  |  |  |
| Follicular | 0.232 | [0.091, 0.374] | 3.226 | **0.001** |  |  |  |
| Ovulatory | 0.138 | [-0.019, 0.295] | 1.725 | 0.085 |  |  |  |
| Mid-luteal | 0.107 | [-0.029, 0.242] | 1.548 | 0.122 |  |  |  |
| Late luteal | [Reference] |  |  |  |  |  |  |
| The multilevel models include fixed and random intercepts. 95% CI, 95% confidence interval; β, regression coefficient. p < 0.05 in bold | | | | | | | |

| **Supplementary Table 3.** Progesterone-to-estradiol ratio in naturally cycling females with alcohol use disorder (AUD): association with menstrual cycle phases | | | | | | | |
| --- | --- | --- | --- | --- | --- | --- | --- |
|  | β | 95% CI | t | p | F | df1, df2 | p |
| N[cases] = 46  N[observations] = 69 |  |  |  |  |  |  |  |
| Time of blood collection | 4.203 | [-1.659, 10.065] |  |  | 2.054 | (1, 62) | 0.157 |
| Cycle phases |  |  |  |  | 5.333 | (4, 45) | **< 0.001** |
| Menstrual | -61.051 | [-106.584, -15.517] | -2.689 | 0.010 |  |  |  |
| Follicular | -78.447 | [-120.868, -36.027] | -3.718 | **< 0.001** |  |  |  |
| Ovulatory | -71.851 | [-116.961, -26.741] | -3.201 | 0.002 |  |  |  |
| Mid-luteal | -19.880 | [-60.684, 20.924] | -0.989 | 0.329 |  |  |  |
| Late luteal | [Reference] |  |  |  |  |  |  |
| The multilevel models include fixed and random intercepts. 95% CI, 95% confidence interval; β, regression coefficient; Progesterone-to-estradiol ratio, [progesterone (ng/mL) * 1,000 (pg/ng)] / [estradiol (pg/mL)] ratio. p < 0.05 in bold | | | | | | | |

| **Supplementary Table 4.** Loss of control over alcohol consumption in males with alcohol use disorder (AUD): AUD severity-specific association with the progesterone-to-estradiol ratio | | | | | | | |
| --- | --- | --- | --- | --- | --- | --- | --- |
|  | β | 95% CI | t | p | F | df1, df2 | p |
| N[cases] = 256  N[observations] = 13,509 |  |  |  |  |  |  |  |
| AUD severity |  |  |  |  | 11.819 | (2, 305) | **< 0.001** |
| Severe | 0.666 | [0.389, 0.944] | 4.728 | **< 0.001** |  |  |  |
| Moderate | 0.327 | [-0.097, 0.556] | 2.806 | **0.005** |  |  |  |
| Mild | [Reference] |  |  |  |  |  |  |
| Weekend days versus weekdays |  |  |  |  | 13.845 | (1, 13252) | **< 0.001** |
| Weekend days | 0.044 | [0.021, 0.068] |  |  |  |  |  |
| Weekdays | [Reference] |  |  |  |  |  |  |
| No lockdown versus lockdown |  |  |  |  | 0.168 | (1, 13385) | 0.682 |
| No lockdown | 0.010 | [-0.038, 0.058] |  |  |  |  |  |
| Lockdown | [Reference] |  |  |  |  |  |  |
| Progesterone-to-estradiol ratio | 0.003 | [-0.001, 0.007] |  |  | 8.507 | (1, 13377) | **0.004** |
| Time of blood collection | -0.027 | [-0.037, -0.017] |  |  | 27.906 | (1, 12907) | **< 0.001** |
| Progesterone-to-estradiol ratio-by-AUD severity |  |  |  |  | 6.959 | (2, 13376) | **< 0.001** |
| Severe | -0.009 | [-0.016, -0.003] | -2.772 | **0.006** |  |  |  |
| Moderate | -0.010 | [-0.016, -0.004] | -3.405 | **< 0.001** |  |  |  |
| Mild | [Reference] |  |  |  |  |  |  |
| The multilevel models include fixed and random intercepts. 95% CI, 95% confidence interval; β, regression coefficient; Progesterone-to-estradiol ratio, [progesterone (ng/mL) * 1,000 (pg/ng)] / [estradiol (pg/mL)] ratio. p < 0.05 in bold | | | | | | | |

| **Supplementary Table 5.** Intensity of alcohol craving in males with alcohol use disorder (AUD): AUD severity-specific association with the progesterone-to-estradiol ratio (interaction effects model) and overall association with the progesterone-to-estradiol ratio (main effects model) | | | | | | | | |
| --- | --- | --- | --- | --- | --- | --- | --- | --- |
|  | β | | 95% CI | t | p | F | df1, df2 | p |
| N[cases] = 256  N[observations] = 8,107 | |  |  |  |  |  |  |  |
| AUD severity |  | |  |  |  | 14.708 | (2, 363) | **< 0.001** |
| Severe | 0.915 | | [0.079, 1.250] | 5.362 | **< 0.001** |  |  |  |
| Moderate | 0.396 | | [0.116, 0.676] | 2.779 | **0.006** |  |  |  |
| Mild | [Reference] | |  |  |  |  |  |  |
| Weekend days versus weekdays |  | |  |  |  | 26.425 | (1, 7851) | **< 0.001** |
| Weekend days | 0.106 | | [0.066, 0.147] |  |  |  |  |  |
| Weekdays | [Reference] | |  |  |  |  |  |  |
| No lockdown versus lockdown |  | |  |  |  | 4.753 | (1, 7987) | **0.029** |
| No lockdown | -0.094 | | [-0.178, -0.009] |  |  |  |  |  |
| Lockdown | [Reference] | |  |  |  |  |  |  |
| Progesterone-to-estradiol ratio | 0.006 | | [-0.000, 0.013] |  |  | 0.001 | (1, 7832) | 0.970 |
| Progesterone-to-estradiol ratio-by-AUD severity |  | |  |  |  | 2.733 | (2, 7821) | 0.065 |
| Severe | -0.013 | | [-0.024, -0.002] | -2.323 | 0.020 |  |  |  |
| Moderate | -0.006 | | [-0.016, 0.004] | -1.207 | 0.227 |  |  |  |
| Mild | [Reference] | |  |  |  |  |  |  |
| Time of blood collection | -0.008 | | [-0.024, -0.002] |  |  | 0.945 | (1, 7016) | 0.331 |
| AUD severity |  | |  |  |  | 11.958 | (2, 246) | **< 0.001** |
| Severe | 0.748 | | [0.443, 1.053] | 4.828 | **< 0.001** |  |  |  |
| Moderate | 0.316 | | [0.067, 0.566] | 2.495 | **0.013** |  |  |  |
| Mild | [Reference] | |  |  |  |  |  |  |
| Weekend days versus weekdays |  | |  |  |  | 26.211 | (1, 7853) | **< 0.001** |
| Weekend days | 0.106 | | [0.065, 0.146] |  |  |  |  |  |
| Weekdays | [Reference] | |  |  |  |  |  |  |
| No lockdown versus lockdown |  | |  |  |  | 4.863 | (1, 7998) | **0.027** |
| No lockdown | -0.095 | | [-0.179, -0.011] |  |  |  |  |  |
| Lockdown | [Reference] | |  |  |  |  |  |  |
| Progesterone-to-estradiol ratio | 0.001 | | [-0.003, 0.005] |  |  | 0.264 | (1, 7884) | 0.608 |
| Time of blood collection | -0.007 | | [-0.024, 0.009] |  |  | 0.757 | (1, 7047) | 0.384 |
| The multilevel models include fixed and random intercepts. 95% CI, 95% confidence interval; β, regression coefficient; Progesterone-to-estradiol ratio, [progesterone (ng/mL) * 1,000 (pg/ng)] / [estradiol (pg/mL)] ratio. p < 0.05 in bold | | | | | | | | |

# **Non-author collaborators of the ReCode Consortium[6, 7]**

Nadja S. Bahr, Felix Bermpohl, Claudia Ebrahimi, Maria Garbusow, Marie Heigert, Christine Heim, Stefanie Kunas, Shuyan Liu, Patricia Pelz, Carlotta Riemerschmid, Nina Romanczuk-Seiferth, Annika Rosenthal, Andreas Ströhle, Heiner Stuke, Henrik Walter, Friederike Wedemeyer, and Julia Wenzel, at Charité–Universitätsmedizin Berlin; Alvaro Aguilera, Viktoria Arndt, Matthew Belanger, Christian Beste, Hao Chen, Tanja Endraß, Pascale C. Fischbach, Sasha Frölich, Filippo Ghin, Charlotte M. Großkopf, Angela Hentschel, Stefan Kiebel, Clemens Kirschbaum, Michael Marxen, Wolfgang E. Nagel, Maximilian Pilhatsch, Johannes Steffen, Jens Strehle, Sarah Schwöbel, Ann-Kathrin Stock, and Gesine Wieder, at Technische Universität Dresden; Acelya Aslan, Herta Flor, Gabriela Gan, Marvin Ganz, Sarah Gerhardt, Damian Karl, Peter Kirsch, Mathieu Pinger, Marcella Rietschel, Kristina Schwarz, Sabine Vollstädt-Klein, Franziska Weiss, Alfred Wieland, Judith Zaiser, and Sina Zimmermann, at Central Institute of Mental Health Mannheim; and Friederike Deeken, at the University of Potsdam.

# **References**

1.	American Psychiatric Association. *Diagnostic and Statistical Manual of Mental Disorders (5th ed.)* 2013. Washington, DC: Author.

2.	Pierl C, Rabstein S, Harth V, Brüning T, Brauch H, Fischer H, et al. Menopausal status and factors affecting cessation of menses in the region of Bonn, Germany. Open Medicine. 2007;2:190-202.

3.	Hampson E. A brief guide to the menstrual cycle and oral contraceptive use for researchers in behavioral endocrinology. Horm Behav. 2020;119:104655.

4.	Deeken F, Reichert M, Zech H, Wenzel J, Wedemeyer F, Aguilera A, et al. Patterns of alcohol consumption among individuals with alcohol use disorder during the COVID-19 pandemic and lockdowns in Germany. JAMA Netw Open. 2022;5:e2224641.

5.	Hoffmann S, Gerhardt S, Mühle C, Reinhard I, Reichert D, Bach P, et al. Associations of menstrual cycle and progesterone-to-estradiol ratio with alcohol consumption in alcohol use disorder: a sex-separated multicenter longitudinal study. Am J Psychiatry. 2024;181:445-456.

6.	Heinz A, Kiefer F, Smolka MN, Endrass T, Beste C, Beck A, et al. Addiction Research Consortium: Losing and regaining control over drug intake (ReCoDe)—from trajectories to mechanisms and interventions. Addict Biol. 2020;25:e12866.

7.	Spanagel R, Bach P, Banaschewski T, Beck A, Bermpohl F, Bernardi RE, et al. The ReCoDe addiction research consortium: Losing and regaining control over drug intake-Findings and future perspectives. Addict Biol. 2024;29:e13419.
